# Supplementary figures and images for: B-cell epitope discovery: The first protein flexibility-based algorithm–Zika virus conserved epitope demonstration
Source: PLoS One. 2023 Mar 15;18(3):e0262321. doi: 10.1371/journal.pone.0262321 (PMC10016673; doi:10.1371/journal.pone.0262321)

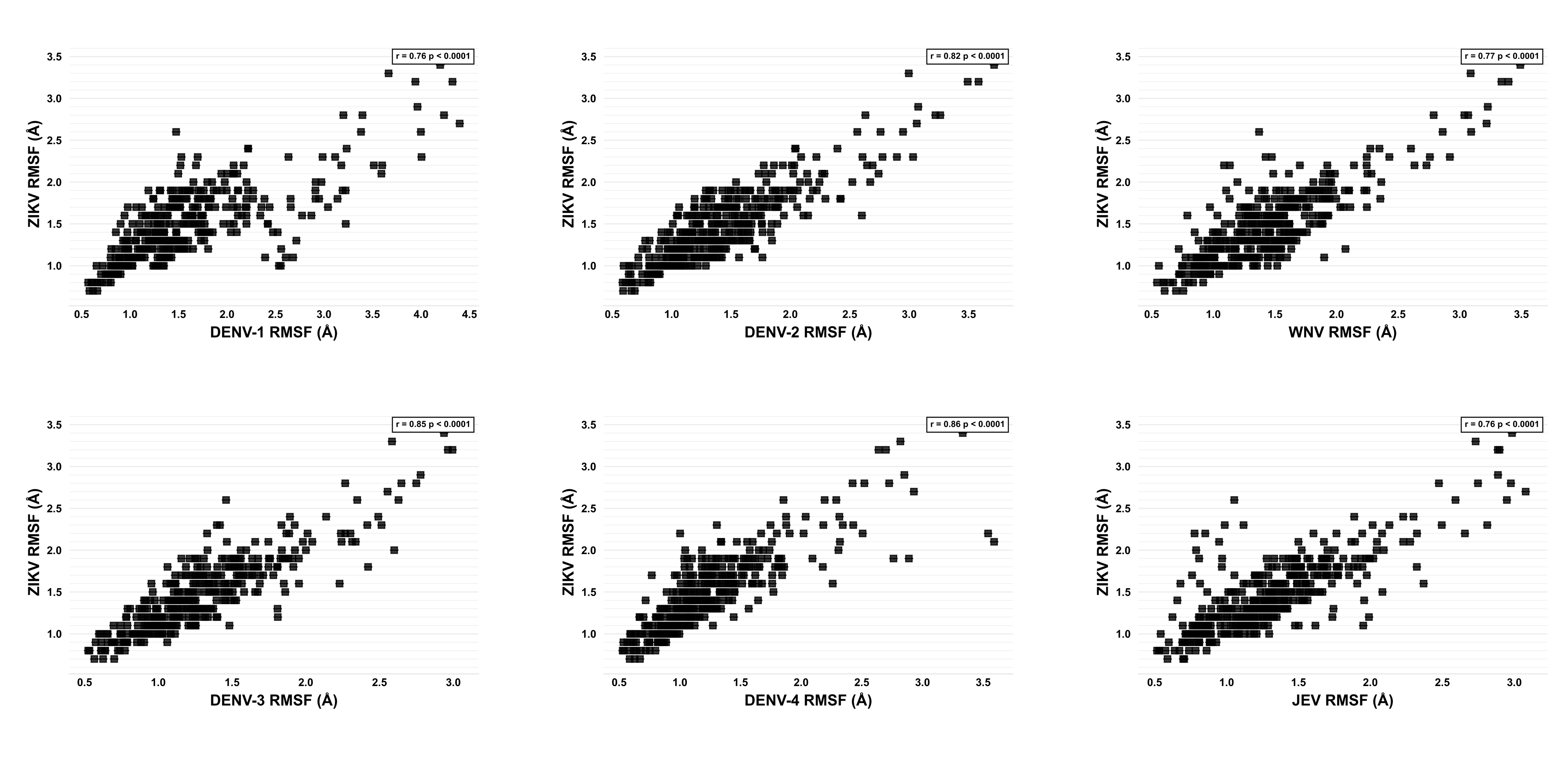

Supplement: S1 Fig — Correlation plots for ZIKV isolated protein RMSF vs. dengue serotype 1 (DENV-1) isolated protein RMSF, dengue serotype 2 (DENV-2) isolated protein RMSF, dengue serotype 3 (DENV-3) isolated protein RMSF, dengue serotype 4 (DENV-4) isolated protein RMSF, West Nile virus (WNV) isolated protein RMSF, and Japanese encephalitis virus (JEV) isolated protein RMSF are shown. Isolated protein refers to a flavivirus envelope protein (with a transmembrane region truncation). Spearman rho (r) and p-values are also shown. (TIF) [file pone.0262321.s001.tif]

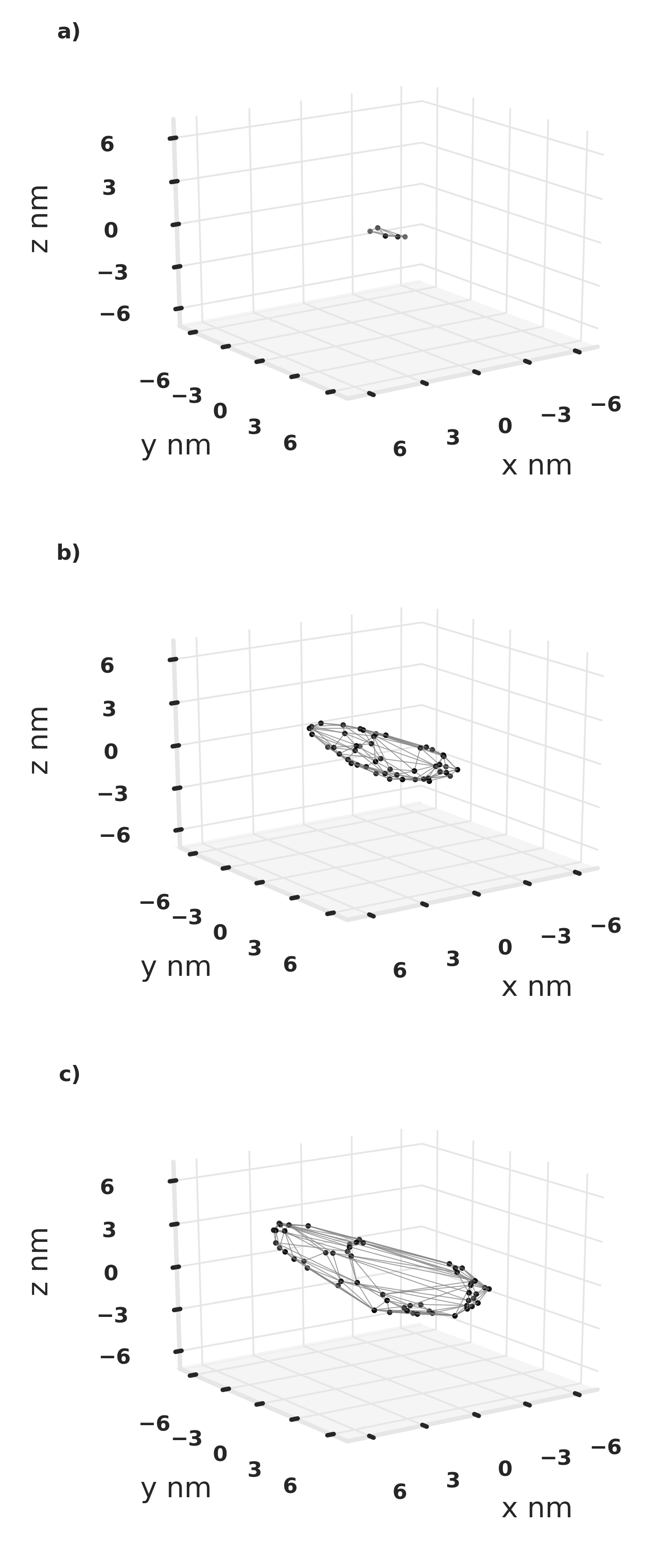

Supplement: S2 Fig — The a) lowest scoring (most inner) and b) median scoring (middle) and c) highest scoring (most outer) isolated protein residue center of masses obtained from convex hull analysis are shown. Isolated protein refers to the ZIKV envelope protein. (TIF) [file pone.0262321.s002.tif]

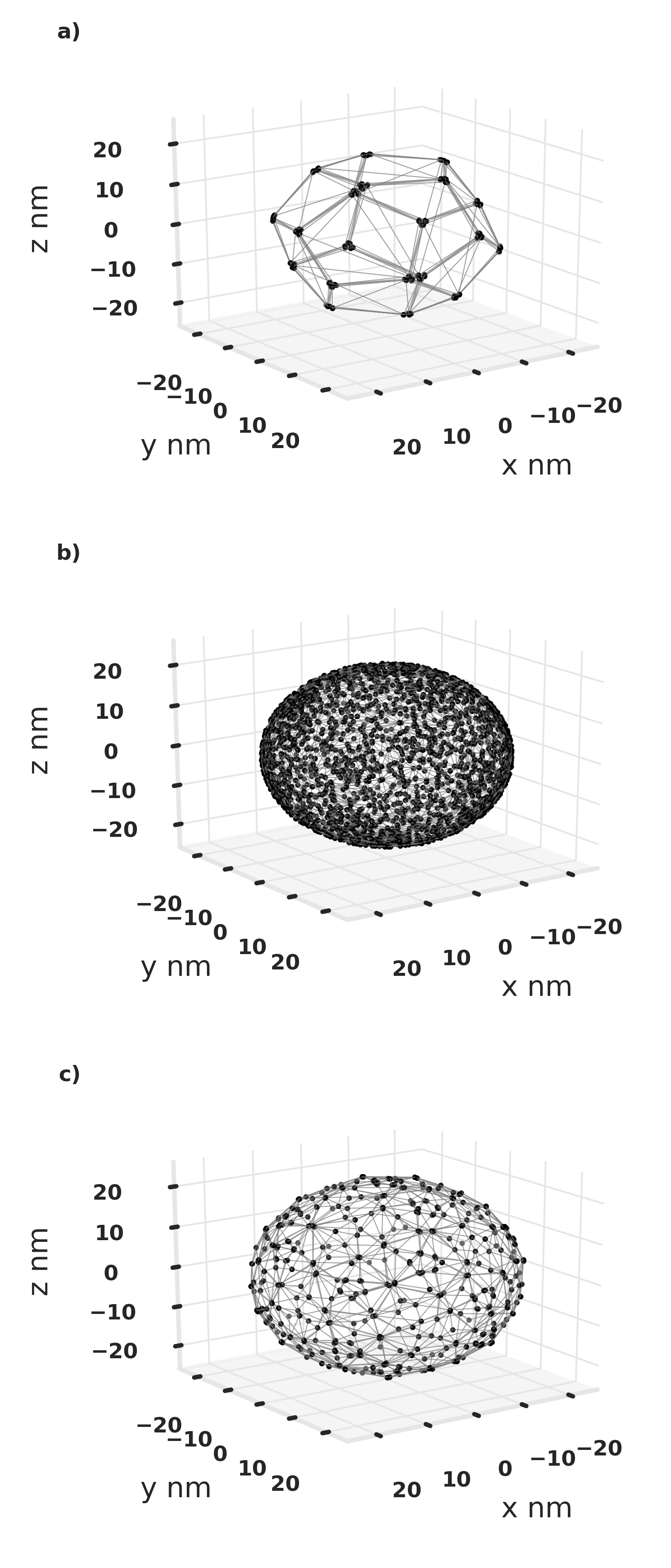

Supplement: S3 Fig — The a) lowest scoring (most inner) and b) median scoring (middle) and c) highest scoring (most outer) VLP protein residue center of masses obtained from convex hull analysis are shown. VLP protein refers to a ZIKV envelope protein (with a transmembrane region truncation) which comprises the VLP (a hollow protein cage). (TIF) [file pone.0262321.s003.tif]
