# Supplementary material for: B-cell epitope discovery: The first protein flexibility-based algorithm–Zika virus conserved epitope demonstration
Source: PLoS One. 2023 Mar 15;18(3):e0262321. doi: 10.1371/journal.pone.0262321 (PMC10016673; doi:10.1371/journal.pone.0262321)
Supplement: S1 Table — Metrics are ordered from the top to bottom in terms of highest ROCAUC and PRAUC product. Spearman rho (r) and p-values are shown for associations between isolated protein convex hull scores (hmon) vs. Epitopia scores (opia), ElliPro scores (epro), isolated protein RMSF (fmon), VLP protein convex hull scores (hvlp), temperature factors (tfac), DiscoTope scores (dtope), VLP protein RMSF (fvlp), BEpro scores (bpro), cons-PPISP scores (ppisp), partially isolated protein RMSF (fmon*), and IUPred scores (iupred). The primary type of structural information utilized for each metric is shown under the heading titled Type (solvent accessible surface area (SASA), protein flexibility (RMSF), and sequence information (SEQ)). (PDF) [file pone.0262321.s004.pdf]

Supplementary Table 1. The individual epitope benchmarking scheme.

Metric	ROCAUC	±	PRAUC	±	Rho	p	Type
**hmon**	0.71	0.16	0.22	0.13	---	---	SASA
**opia**	0.67	0.12	0.19	0.14	0.61	< 0.01	SASA
**epro**	0.72	0.19	0.16	0.18	0.88	< 0.01	SASA
**fmon***	0.69	0.17	0.15	0.20	0.73	< 0.01	RMSF
**fmon**	0.70	0.17	0.13	0.14	0.48	< 0.01	RMSF
**hvlp**	0.65	0.21	0.11	0.11	0.43	< 0.01	SASA
**bpro**	0.59	0.09	0.11	0.08	0.51	< 0.01	SASA
**tfac**	0.58	0.17	0.09	0.09	0.59	< 0.01	RMSF
**dtope**	0.65	0.14	0.08	0.06	0.61	< 0.01	SASA
**fvlp**	0.59	0.21	0.08	0.06	0.45	< 0.01	RMSF
**ppisp**	0.54	0.16	0.05	0.03	0.11	0.03	SASA
**iupred**	0.52	0.11	0.05	0.04	0.08	0.12	SEQ

S1 Table. Epitope discovery performance benchmarking on individual epitopes. Metrics are ordered from the top to bottom in terms of highest ROCAUC and PRAUC product. Spearman rho (r) and p-values are shown for associations between isolated protein convex hull scores (hmon) vs. Epitopia scores (opia), ElliPro scores (epro),

isolated protein RMSF (fmon), VLP protein convex hull scores (hvlp), temperature factors (tfac), DiscoTope scores (dtope), VLP protein RMSF (fvlp), BEpro scores (bpro), cons-PPISP scores (ppisp), partially isolated protein RMSF (fmon*), and IUPred scores (iupred). The primary type of structural information utilized for each metric is shown under the heading titled Type (solvent accessible surface area (SASA), protein flexibility (RMSF), and sequence information (SEQ)).
