# Supplementary material for: B-cell epitope discovery: The first protein flexibility-based algorithm–Zika virus conserved epitope demonstration
Source: PLoS One. 2023 Mar 15;18(3):e0262321. doi: 10.1371/journal.pone.0262321 (PMC10016673; doi:10.1371/journal.pone.0262321)
Supplement: S2 Table — Isolated protein flexibility of seven flavivirus structures is examined for epitope discovery performance against the top seven ZIKV-aligned, conserved flavivirus epitopes. Metrics are ordered from the top to bottom in terms of highest ROCAUC and PRAUC product. Spearman rho (r) and p-values are shown for associations between ZIKV isolated protein RMSF (zikv) vs. Japanese encephalitis virus isolated protein RMSF (jev), dengue serotype 2 isolated protein RMSF (denv2), West Nile virus isolated protein RMSF (wnv), dengue serotype 4 isolated protein RMSF (denv4), dengue serotype 3 isolated protein RMSF (denv3), and dengue serotype 1 isolated protein RMSF (denv1). (PDF) [file pone.0262321.s005.pdf]

**Supplementary Table 2. Flavivirus flexibility comparison.**

| Metric | ROCAUC | ± | PRAUC | ± | Rho | p |
|---|---|---|---|---|---|---|
| **zikv** | 0.72 | 0.07 | 0.48 | 0.10 | --- | --- |
| **jev** | 0.64 | 0.18 | 0.52 | 0.21 | 0.76 | < 0.01 |
| **denv4** | 0.72 | 0.15 | 0.44 | 0.18 | 0.86 | < 0.01 |
| **wnv** | 0.62 | 0.17 | 0.50 | 0.16 | 0.77 | < 0.01 |
| **denv1** | 0.61 | 0.16 | 0.47 | 0.19 | 0.76 | < 0.01 |
| **denv2** | 0.61 | 0.18 | 0.46 | 0.10 | 0.82 | < 0.01 |
| **denv3** | 0.60 | 0.16 | 0.43 | 0.10 | 0.85 | < 0.01 |

**S2 Table. Flavivirus isolated protein flexibility: conserved epitope discovery performance benchmarking.** Isolated protein flexibility of seven flavivirus structures is examined for epitope discovery performance against the top seven ZIKV-aligned, conserved flavivirus epitopes. Metrics are ordered from the top to bottom in terms of highest ROCAUC and PRAUC product. Spearman rho (r) and p-values are shown for associations between ZIKV isolated protein RMSF (zikv) vs. Japanese encephalitis virus isolated protein RMSF (jev), dengue serotype 2 isolated protein RMSF (denv2), West Nile virus isolated protein RMSF (wnv), dengue serotype 4 isolated protein RMSF (denv4), dengue serotype 3 isolated protein RMSF (denv3), and dengue serotype 1 isolated protein RMSF (denv1).
