# Supplementary material for: B-cell epitope discovery: The first protein flexibility-based algorithm–Zika virus conserved epitope demonstration
Source: PLoS One. 2023 Mar 15;18(3):e0262321. doi: 10.1371/journal.pone.0262321 (PMC10016673; doi:10.1371/journal.pone.0262321)
Supplement: Attachment — Submitted filename: Biner.Protein.Flexibility.Rebuttal.Letter.PLOS.docx [file pone.0262321.s007.docx]

Overall:

**We would like to thank the reviewers for their thoughtful feedback.**

Reviewer #1:
1. Page 17, line 374, equation 1: The expression for RMSF seems to indicate that each RMSF is calculated over the backbone atoms; exactly what atoms are considered “backbone”, are hydrogens included in the calculation?

**The “backbone” group setting within GROMACS was used which includes** **only N, Cα and C atoms. This has been added to the *Methods*.**

2. A few citations need to be added to introduce a general audience to the field of whole capsid simulations. Prof. Ortoleva is a leader in the field, but the manuscript would benefit itself by putting itself in the context of this emerging field.

**An introduction to molecular dynamics simulations of virus-like particles is now included in the *Introduction*.**

3. Solvation of the system is introduced casually; however, the authors do not provide evidence that the internal pressure of the system is equilibrated. This is common practice in the field of simulating entire viral capsids (e.g., Andoh et al. JCP, Perilla and Schulten. Nat. Comm., Reddy and Samson, Structure).

**We now discuss virus-like particle simulation equilibration in terms of water exchange rates in the *Results*. Thank you for this suggestion.**

4. It seems that ZIKV was simulated in the absence of the lipids, what changes would the author expect if the membrane were simulated explicitly. Would the extension of their analysis to other viruses still hold?

**We suspect simulation of the membrane (transmembrane envelope protein region and M protein) may reduce the permeability of the VLP and dampen the flexibility of the proteins in the VLP. Overall, we do not believe the absence of lipids would influence the extension of our flexibility-based method to other viruses, as we show the VLP proteins have higher rigidity than the isolated protein even without the extra components. This is briefly discussed when explaining water exchange rates for the VLP in the *Epitope discovery performance benchmarking and rationale for a new method* section of the *Results*.**

5. A main concern with the present study is reproducibility; it is understandable that trajectories are too large to deposit, however trajectories of only the backbone atoms (used for most analysis) could be posted to a library-sponsored repository. The authors are also encouraged to upload their code and methods to GitHub.

**We have reached out to a library sponsored repository for storing our simulation files. They have asked us to touch base around April 20th when they have more staff available for large uploads.**

**We would like to release a source eventually; However, the code for epitope prediction needs a reorganization before the source would be helpful to other researchers. Because of this, we spent a large amount of time detailing the method in the *Methods* – to ensure other researchers could replicate our method regardless of when a source is available or their experience with a specific coding language.**

Reviewer #2:
1. The benchmarking is based on a limited number of (highly conserved) epitopes and it is not clear if the method is indeed generalizable to other systems.

**We now discuss the generalizability of the flexibility-based method (in more depth) in the *Results*.**

**1) We explain conserved epitopes (by definition) are of especially high value in vaccine design (compared with non-conserved epitopes) because they generalize across many pathogens (Table 3).**

**2) We explain how our method likely generalizes across enveloped pathogens by quantifying the flexibility of seven flaviviruses and explaining how our results mesh with previous reports of HIV antibody maturation-flexibility associations.**

**3) We now show our flexibility-based method performs better than other methods at pinpointing highly conserved human papillomavirus epitopes (a non-enveloped system).**

2. Manuscript is hard to follow at times. For example, p25 step 2 says the epitope dataset was split into test (20%) and training (80%). It’s not clear if only 7 epitopes or all 38 epitopes are used, or a different dataset was used (for comparison, Epitopia uses 66 non-redundant validated epitopes derived from co-crystal structure + 194 non-redundant validated epitopes derived from antigen sequences). In the methods, the definition of RMSF is given but how exactly it is used in the benchmarking is not clear.

**We separately benchmarked structural metrics against 1) all 39 ZIKV-aligned epitopes (the *Epitope discovery performance benchmarking and rationale for a new method* section of the Results) and 2) the seven most conserved ZIKV aligned epitopes (the *Conserved epitope discovery performance benchmarking and the flexibility-based model* section of the Results).**

**We now specify in the text whenever we use test and train dataset splitting for linear combination.**

**Although the Epitopia dataset and server is not online as of writing this response, we think the Epitopia benchmark dataset made no distinction between 1) conserved epitopes and non-conserved epitopes and 2) pathogenic antigens and non-pathogenic antigens, so a comparison with our dataset is difficult to make. Other preexisting datasets also have biases which make comparison with our dataset challenging. For example, the DiscoTope dataset includes epitopes from seventy-six crystal structures, but twenty-nine of the structures (almost half) were variants of lysozyme (a small globular enzyme (1 protein, ~100 residues) as opposed to a large, macromolecular pathogen (180 protein copies, 400+ residues)) (as discussed by Haste Andersen, Pernille, Morten Nielsen, and Ole Lund. "Prediction of residues in discontinuous B‐cell epitopes using protein 3D structures." Protein Science 15.11 (2006): 2558-2567.).**

**(See Answer #1) We now show our flexibility-based method performs better than other methods at pinpointing highly conserved human papillomavirus epitopes (a non-enveloped pathogen).**

**All metrics including RMSF were benchmarked using AUC analysis (the *Epitope discovery performance benchmarking and rationale for a new method* section of the *Methods*). AUC analysis quantifies how well a continuous signal (the RMSF residue profile) ranks a binary outcome (residues in epitopes vs residues outside epitopes).**

3. RMSF of the surface residues may correlate with the solvent accessibility, this study with the limited dataset may lack confidence in the overall approach.

**The correlations between isolated flexibility and the other metrics are shown in Table 2. We now discuss flexibility-solvent accessibility correlations (in more depth) in the *Epitope discovery performance benchmarking and rationale for a new method* section of the *Results*. Importantly, 1) we note in the text accessibility to antibody-antigen is likely a prerequisite an epitope’s existence, 2) we discuss why it might be advantageous for a conserved epitope to have partial or transient accessibility, and 3) we mention existence of a subset of epitopes, termed “cryptic” epitopes, which can appear to have low solvent accessible surface area on static protein structures.**

**(See Answer to #1) We now show our flexibility-based method performs better than other methods at pinpointing highly conserved human papillomavirus epitopes (a non-enveloped system).**

4. Minor: the authors can discuss why only contiguous, linear sequences are predicted. Also, it will be better to include a table with the sequence details (in addition to the residue), e.g. in page 16,17. All, predicted epitopes could be presented with the most conserved highlighted.

**A table with predicted epitopes is now included. We now briefly discuss the continuity of the epitope predictions in the *Conserved epitope organization and epitope discovery demonstration* section of the Results.**

Reviewer #3:

Precision-Recall Area Under the Curve:

1. In Table 1, please include a column explaining the info each prediction software utilizes, e.g., IUPred takes in sequence info.

**This is a great suggestion. The primary structural information utilized with each metric are now included in Tables 1-3.**

2. Please provide the raw datasets (compiled ZIKV-aligned epitopes) used for the analyses in creating the Table 1.

**The raw ZIKV-aligned epitope dataset is now included as Supplementary .csv (S1 Dataset). The Supplementary .csv is now mentioned at the beginning of the *Epitope discovery performance benchmarking and rationale for a new method* section of the *Results.***

3. I believe baseline is not the correct word here. Moreover, ‘baseline’ value for ROCAUC and PRAUC is different depending on the input (compiled/isolated protein). So, I suggest mentioning this ‘baseline’ value wherever applicable in the Results/Discussion section. State either in percentage or in ratio throughout this section. Do not use interchangeably. This reduces confusion.

**We now refer to this value as the value of no discrimination. The value is now shown as a ratio (in decimal format) for each AUC value discussed. We have also added a small paragraph explaining what the value of no discrimination is and why we provide it at the beginning of the *Epitope discovery performance benchmarking and rationale for a new method* section of the *Results*.**

**The value of no discrimination is always 0.50 for ROCAUC analysis. For PRAUC analysis on a compilation of epitopes, each AUC calculation maintains the same value of no discrimination as the proportion of epitope to non-epitope residues in the dataset is maintained for each split of the dataset using stratification (The *Epitope discovery performance benchmarking and rationale for a new method* section of the *Methods*) (Fig 5). For benchmarking on individual epitopes, we provide the average value of no discrimination.**

4. Also, if you include metric (s) from one more prediction tool, the ‘baseline’ value may change as well. You cannot call it a threshold either. So, I am not in favour of this value.

**We do not believe the value of no discrimination changes when metric(s) from multiple prediction tools are combined, as the value of no discrimination is dependent on the epitope dataset: 0.50 for ROCAUC analysis and the proportion of epitope residues to non-epitope residues in the dataset for PRAUC analysis.**

**Also, we believe this value is important to the manuscript – as the value of no discrimination facilitates comparison between ROCAUC and PRAUC values (as shown in the *Epitope discovery performance benchmarking and rationale for a new method* section of the *Results*).**

5. The authors always mention isolated protein or compilation of epitopes. Nowhere in the Results/Discussion section they mention that they talk about envelope protein. I see only one sentence in the Materials and Methods part. Even in the Figure legends, the authors should state the source of the epitope (e.g., envelope protein).

**We have included a small paragraph in the *Epitope discovery performance benchmarking and rationale for a new method* section of the Results where we let the audience know we mean envelope protein when we refer to a ZIKV or flavivirus protein. This way we 1) can reduce clutter within the text (as we mention the ZIKV protein numerous times throughout the text) and 2) will hopefully not confuse our audience who have yet to know what an envelope protein is.**

6. The authors mention VLP protein. Is it a sphere (vesicle) decorated with only envelope protein or it also has membrane protein?

**A sentence has been added to the beginning of the *Epitope discovery performance benchmarking and rationale for a new method* section of the Results explaining what the VLP model is. We also discuss the VLP in the *Model preparation and molecular dynamics simulations* section of the *Methods* and have also depicted convex hulls of the VLP in S3 Fig.**

7. I feel the authors have used ‘epitope residues’ and ‘epitopes’ interchangeably. They must correct this at certain places to avoid confusion.

**We make a purposeful distinction between epitope residues (residues which contact an antibody) and an epitope (a specific grouping of residues contacted by an antibody). This distinction is important as AUC analysis identifies epitope residues, but not epitopes. Epitope residues identified via AUC analysis need to be clustered for the identification of full epitopes.**

8. In Fig 1 legend, the authors mention ‘a single VLP protein’. What do they mean by that? It is not clear.

**See Answer #6.**

Conserved epitope discovery performance benchmarking:

1. The authors have used flavivirus and ZIKV interchangeably. Since they also talk about DENV, JEV and WNV, it brings confusion at certain places.

**We have attempted to add clarity to this distinction.**

2) The authors should include their definition for ‘isolated protein’ way before than stating in the middle of the manuscript.

**We now explain the term isolated protein and partially isolated protein much earlier in the *Epitope discovery performance benchmarking and rationale for a new method* section of the *Results*. We have also included a little more discussion about partially isolate protein flexibility.**

3) In Fig 3, I would also like to see a graph with SASA values plotted

**Fixed.**

Conserved epitope organization and epitope discovery demonstration:

1) Line 283: Is seven correct?

**Yes.**

2) In Table 3, please also include a column with virus names.

**Fixed**

3) In Fig 4, ‘…epitopes are shown as enlarged spheres (yellow)’. I would rather say epitopes are represented in yellow.

**Fixed.**

Methods:

Please rearrange the flow (subheadings) as per your Results/Discussion section.

**We have organized our *Methods* subheadings in a way which mirrors the flow of the overall method: 1) modeling and simulation, 2) quantification of structural metrics, 3) quantification of the epitope dataset, 4) performance benchmarking, and 5) epitope organization and prediction. We believe this is the most logical organization.**

In S1 Fig., please mention the definition for the isolated the protein. Including, e.g., envelope protein.

**Fixed.**

In S1 Fig., the authors show flexibility correlation plots between ZIKV and other flaviviruses. Similar observation has been earlier made by Palanisamy et al. (Protein backbone flexibility pattern is evolutionarily conserved in the Flaviviridae family: a case of NS3 protease in Flavivirus and Hepacivirus), although they showed it for protease and polymerase. The authors should discuss about it.

**Fixed. Thank you for bringing this to our attention.**

In S2 Fig., likewise

**Fixed.**

In S3 Fig., likewise

**Fixed.**

General:

The authors make a strong statement in this manuscript; protein flexibility is shown to outperform solvent accessible surface area as an epitope discovery metric. The authors should also show for other viruses (e.g. DENV), at least one more depending on the availability of the datasets, whether it is the case.

**“Cryptic” epitopes (those which appear to be solvent inaccessible on static strictures) have been characterized structurally. Based on the analysis of our data (along with the previous literature), we believe stabilization of isolated virus protein regions upon virus assemble (or maturation) is a straightforward and effective method for transiently protecting vulnerable regions of pathogens from the immune system. Additionally, we have included data on conserved human papillomavirus epitopes, a system which is non-enveloped.**
