# Supplementary material for: B-cell epitope discovery: The first protein flexibility-based algorithm–Zika virus conserved epitope demonstration
Source: PLoS One. 2023 Mar 15;18(3):e0262321. doi: 10.1371/journal.pone.0262321 (PMC10016673; doi:10.1371/journal.pone.0262321)
Supplement: Attachment — Submitted filename: Biner.Protein.Flexibility.Rebuttal.Letter.PLOS.2.docx [file pone.0262321.s008.docx]

Reviewer #1:

**1) The ZIKV protein simulations were performed without a membrane, even though the transmembrane region is included in the model. The authors explained very well what are the limitations of this set up in the response to the reviewers, but not in the manuscript. I would suggest that they add the same explanation in the main text, e.g. in the Methods ("Model preparation and molecular dynamics simulations" section).**

A paragraph was added to the Discussion and Model Preparation sections.

“Before proceeding, it is important for clarity to address terminology and limitations of the current study. First, because a single flavivirus gene product – the envelope protein[31] – forms the basis for epitope discovery performance benchmarking here, this protein is referred to as the ZIKV (or flavivirus) protein when isolated and the ZIKV VLP protein when assembled into a VLP (for more detail, see the Model preparation and molecular dynamics simulations section of the Methods). Second, because the simulated ZIKV VLP is a reduced representation of the mature ZIKV (e.g. simulated in the absence of structural components present in the mature ZIKV; S3 Fig), metrics derived from VLP simulation may miss structural aspects of the infectious virus.”

“To build a soluble ZIKV VLP simulation starting structure, protein coordinates from ZIKV chains A, C, and E (residues 1-402) and an associated symmetry matrix (taken from PDB ID: 5IRE) were used. The viral membrane was excluded from VLP simulation starting structure to reduce the complexity of the system. It is hypothesized here simulation of the VLP with membrane could 1) reduce the permeability of the VLP and 2) dampen flexibility of the proteins in the VLP, without radically changing the relative positions of maxima and minima on the flexibility residue profile (Fig 3).”

**2) Maybe I overlooked it but I could not find the duration/length of the simulations. Can the authors indicate how many nanoseconds they ran for each system?**

The simulation duration is now mentioned in the Methods and Discussion.

“Using GROMACS 5.0.4,[66] explicit-solvent (TIP3P)[67] MD simulations were run on the flavivirus proteins and ZIKV VLP to a local structural equilibrium on the Indiana University Big Red II and III super-computers (Fig 1). Attainment of local structural equilibrium was assessed as a steady time-average in backbone atom root-mean-square deviation (RMSD) relative to the starting configuration.[20] The majority of simulations were run for ~50 ns.”

“No difference in performance was observed between VLP protein convex hull scores quantified from the cryo-EM reconstruction vs those quantified from the VLP MD simulation. Overall, these results 1) join growing support for the hypothesis accessibility to antibody binding is one prerequisite for an epitope’s existence,[5, 33, 39] 2) suggest morphological diversity may challenge our assumptions as to which regions of a pathogen are accessible to antibody binding, and 3) suggest the simulated VLP sampled con-formations which were a) relatively similar to the cryo-EM reconstruction and b) stable enough for flexibility estimation by 50 ns.”

**3) The authors mentioned "cryptic epitopes", which exhibit low solvent accessible surface area in static structures. Have they investigated the time evolution of the SASA of these cryptic pockets along their MD trajectories?**

Convex hull scores of the simulated VLP were quantified and benchmarked.

“Interestingly, despite poor capacity to rank conserved epitopes above all other residues (a ROCAUC mean ~0.10 below the 0.50 value of no discrimination), ZIKV VLP protein convex hull scores derived from the MD simulation performed with relatively high capacity to pinpoint conserved epitope residues (a PRAUC mean above the 0.07 value of no discrimination) suggesting, although VLP convex hull scores performed below average (in terms of conserved epitope discovery), conserved epitopes are less concealed on the simulated VLP than the cryo-EM reconstruction.”

“An analogous process was performed for the simulated ZIKV VLP. VLP coordinates were averaged for each 5 ns equilibrated trajectory segment. Convex hull scores were then 1) quantified for four equilibrated trajectory segments and 2) averaged for each residue over all trajectory segments.”

**4) The authors explained in the response letter that they are negotiating with a repository to store their simulation files. For the sake of the data availability and reproducibility, the authors should include in the manuscript the link to the repository or offer the possibility to obtain the data upon request to the authors.**

All simulations are organized, backed up, and available at: https://scholarworks.iu.edu/dspace/handle/2022/26440.

Reviewer #2:

**1) I know that the systems are very big, but do the authors consider that just 50 ns MD simulations are enough to get stable conformations of the systems under study? Moreover, as the simulations are used to analyze the flexibility properties (RMSF, for example), Did the authors perform replicas in order to make the predictions more robust? These may strengthen all the conclusions.**

Simulation duration, system equilibration, and stable conformations are now mentioned more directly in the Discussion.

“No difference in performance was observed between VLP protein convex hull scores quantified from the cryo-EM reconstruction vs those quantified from the VLP MD simulation. Overall, these results 1) join growing support for the hypothesis accessibility to antibody binding is one prerequisite for an epitope’s existence,[5, 33, 39] 2) suggest morphological diversity may challenge our assumptions as to which regions of a pathogen are accessible to antibody binding, and 3) suggest the simulated VLP sampled conformations which were a) relatively similar to the cryo-EM reconstruction and b) stable enough for flexibility estimation by 50 ns.”

“To better understand protein flexibility-based epitope discovery performance, RMSD equilibration plots and water exchange rates (of simulations) were examined (Fig 1). RMSD equilibration plots show a steady time-average in backbone atom RMSD relative to the starting configuration for both the isolated protein and VLP proteins, as expected for equilibrated systems. A 0.15 Å RMSD standard deviation (on average) was observed for all VLP proteins (analyzed individually) vs. a 0.37 Å RMSD standard deviation observed for the isolated protein (at 300K over the last 20 ns). In terms of water exchange rates, the rate of waters entering and exiting the ZIKV VLP were 122,973 ± 1.344 and 123,105 ± 1,328 per ns (~5% of the total waters in the capsid) respectively (over the last 20 ns of the simulation), providing further evidence the VLP had equilibrated prior to protein flexibility estimation.”

“Comparison of ZIKV VLP water exchange rates with those of a simulation of the mature HIV capsid showed the ZIKV VLP had around two orders of magnitude higher permeability than the mature HIV capsid.[18] Despite relatively high permeability, the VLP maintained its structural integrity – as suggested by similarities in epitope discovery performance between convex hull scores quantified from the simulated ZIKV VLP vs those quantified from the cryo-EM reconstruction (Table 1). Relatively high permeability of the ZIKV VLP could be related to 1) differences between envelope proteins and capsid proteins and 2) the absence of membrane and membrane-associated protein components within the ZIKV VLP.”

1) An additional isolated protein simulation was incorporated into the isolated protein flexibility metric.
2) 10 recently characterized flavivirus epitopes were included in the epitope dataset.
3) A paragraph explaining how the statistical significance of our flexibility-based metrics were enhanced was added to the Methods.

“To enhance statistical significance of isolated ZIKV protein flexibility, RMSF was calculated over structurally equilibrated 5 ns trajectory segments from a simulation at 300 K and a simulation at 310 K. To enhance the statistical significance of ZIKV VLP protein flexibility, mean RMSF amplitudes were calculated for each residue over all proteins, as all 180 proteins which comprise the VLP are quasi-equivalent.[76] An analogous process was performed to quantify HPV RMSF using four equilibrated 2.5 ns trajectory segments.”

**2) I agree with the rationale behind not using the explicit membrane in the ZIKV VLP system, but why not testing the putative effects of the membrane in the isolated systems, and then transfer this information to the ZIKV VLP system?**

We are uncertain as to whether or not the effects of the membrane on the isolated system transfer to the VLP in a straight forward manner, as the VLP proteins are cooperatively linked unlike the isolated protein.

“To build a soluble ZIKV VLP simulation starting structure, protein coordinates from ZIKV chains A, C, and E (residues 1-402) and an associated symmetry matrix (taken from PDB ID: 5IRE) were used. The viral membrane was excluded from VLP simulation starting structure to reduce the complexity of the system. It is hypothesized here simulation of the VLP with membrane could 1) reduce the permeability of the VLP and 2) dampen flexibility of the proteins in the VLP, without radically changing the relative positions of maxima and minima on the flexibility residue profile (Fig 3).”

“Before proceeding, it is important for clarity to address terminology and limitations of the current study. First, because a single flavivirus gene product – the envelope protein[31] – forms the basis for epitope discovery performance benchmarking here, this protein is referred to as the ZIKV (or flavivirus) protein when isolated and the ZIKV VLP protein when assembled into a VLP (for more detail, see the Model preparation and molecular dynamics simulations section of the Methods). Second, because the simulated ZIKV VLP is a reduced representation of the mature ZIKV (e.g. simulated in the absence of structural components present in the mature ZIKV; S3 Fig), metrics derived from VLP simulation may miss structural aspects of the infectious virus.”

**3) Moreover, the data are not available yet. Do the authors have updates regarding data availability? I am afraid that this is fundamental for publication in PLOS One.**

All simulations are organized, backed up, and available at: https://scholarworks.iu.edu/dspace/handle/2022/26440.
